# Mitral Regurgitation: Anatomy, Physiology, and Pathophysiology—Lessons Learned From Surgery and Cardiac Imaging

**DOI:** 10.3389/fcvm.2020.00084

**Published:** 2020-05-29

**Authors:** Yan Topilsky

**Affiliations:** The Department of Cardiology, Tel Aviv Medical Center, Sackler Faculty of Medicine, Tel Aviv University, Tel Aviv-Yafo, Israel

**Keywords:** mitral regurgitation, mitral annulus, papillary muscles, left ventricle, left atrium

## Abstract

The normal mitral valve is a dynamic structure that permits blood to flow from the left atrial (LA) to left ventricle (LV) during diastole and sealing of the LA from the LV during systole. The main components of the mitral apparatus are the mitral annulus (MA), the mitral leaflets, the chordae tendineae, and the papillary muscles (PM) (Figure 1). Normal valve function is dependent on the integrity and normal interplay of these components. Abnormal function of any one of the components, or their interplay can result in mitral regurgitation (MR). Understanding the anatomy and physiology of all the component of the mitral valve is important for the diagnosis, and for optimal planning of repair procedures. In this review we will focus first on normal anatomy and physiology of the different parts of the mitral valve (MA, leaflets, chordae tendineae, and PM). In the second part we will focus on the pathologic anatomic and physiologic derangements associated with different types of MR.

## Mitral Valve Apparatus Anatomy and Physiology

### Mitral Annulus

**Figure 1:**
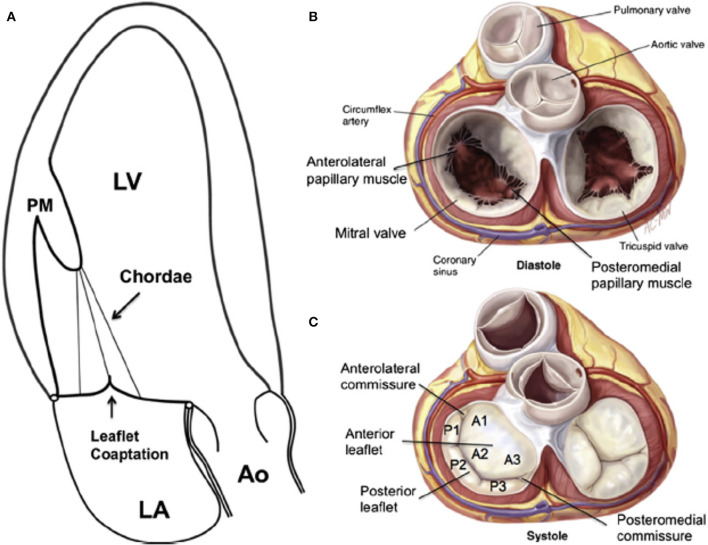
**(A)** Apical long-axis view of the normal heart in systole. There is no tethering, both leaflets are normal, concave toward the LV, and co-aption is in the annular level. **(B)** The open mitral valve in diastole from the atrial view. **(C)** Surgical view of the closed mitral valve in systole showing the anterior “sail-like” leaflets with no indentations vs. the posterior segmented leaflet. A, anterior leaflet; Ao, aorta; LA, left atrium; LV, left ventricle; P, posterior leaflet; PM, papillary muscle. From Carpentier A. Carpentier's reconstructive valve surgery. St. Louis: Saunders/Elsevier; 2010; with permission.

The mitral annulus (MA) is defined by the tissue intersection between the LA, LV, and the mitral leaflets. It is dynamic throughout the cardiac cycle, is made out of parallel collagen fibers and is well-defined histologically. It is non-planar and shaped like a saddle (Figure 1, Supplementary Movie 1) (1, 2). The anterior portion of the MA is continuous with the aortic annulus and constitutes the most atrial part of the saddle shape (2). The posterior part of the MA includes the lowest points of the saddle close to the lateral and medial commissures. Compared with the anterior portion, the posterior MA is not anchored strongly to the neighboring tissue, allowing more free movement during myocardial contraction and relaxation.

The angle between the MA to the aortic annulus changes during the cardiac cycle through the mitral aortic fibrous continuity (3). Recent studies, using 3-D echocardiography (1, 4, 5) have assessed the normal mitral annulus changes over the cardiac cycle. They have showed that variation of annular size throughout diastole is minimal. However, in early-systole, during the iso-volumic contraction period, antero-posterior contraction occurs, resulting in folding across the fixed inter-commissural diameter. This contraction leads to very early-systolic annular area contraction, accentuation of saddle shape, and approximation of anterior and posterior leaflets. In other words, when early-systolic ventricular pressure is still relatively low, leaflet approximation by annular contraction and saddle shape accentuation results occurs even before LV pressure rises, locking the leaflets together. This mechanism may be important in preventing early systolic mitral regurgitation (Supplementary Movie 1). The mechanism for this early saddle-shape accentuation is disputed. Some have postulated it is related to tethering of the anterior annulus to the aortic root combined with apical translation of the “loose” posterior annulus resulting in folding across the inter-commissural axis.

The MA is innervated and supplies blood vessels to the leaflets (6, 7).

Several recent investigations using advanced imaging modalities reported an average mitral annular area of ~10 cm^2^ in healthy subjects (8–10), much larger than the widely believed “normal” mitral annular orifice area of 4–6 cm^2^.

### Mitral Valve Leaflets

**Figure 2:**
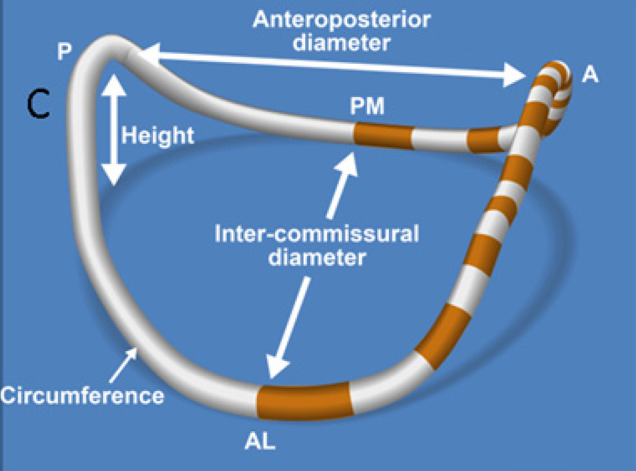
Schematic representation of a 3D reconstructed mitral. annulus. AL, antero-lateral; PM, postero-medial; ML, medial-lateral (intercommisural) diameter; AP, antero-posterior diameter.

The mitral valve leaflets fully open and close up to 3,000,000,000 times throughout a lifetime (11). Despite this high burden, significant mitral valve disease is uncommon in patients younger than 65 years. The mitral valve has anterior and posterior leaflets and variable commissural scallops to occlude medial and lateral gaps (Figure 2). Leaflet tissue is attached to the MA, and the normal tissue length is between 0.5 and 1.0 cm (12). Redundant leaflet tissue is very important for tight leaflet co-aptation and sealing. The normal ratio of mitral annulus to mitral leaflet area is 1.5–2.0, and is vital to prevent significant MR in normal, and even dilated left ventricles (12). The normal atrial surface of the leaflets is smooth. A hydrophilic protein-rich part, called the rough zone, starts ~1 cm from the distal leaflet tips and helps to ensure a perfect seal between the leaflets, termed the co-aptation zone. The ventricular surface of the anterior leaflet is made out of collagen fibers originating from the chordal insertion continuing up to the annulus. There are two types of chordae, the primary and secondary chords. The primary chords insert at the tips of the leaflets, but the secondary chords attach to the leaflets close to the rough zone (13). The anterior mitral leaflet is shaped like a sail, and is anchored to the fibrous portion of the MA. It is continuous with the fibrous tissue of the non-coronary cusp of the aortic valve (Figure 2). It is tightly anchored to the left and right fibrous trigones by collagen fibers (14). It is larger, longer, and thicker than the posterior leaflet (see Figure 2). The anterior leaflet is divided into lateral (A1), central (A2), and medial scallops (A3). However, for the anterior leaflet, this nomenclature does not represent real anatomically distinct structures, and the sub-division is done only to simplify medical communication (Figure 2).

The posterior leaflet is crescentic in shape and has a much longer circumferential connection (≈5 cm) to the MA compared with the anterior leaflet (≈3 cm). However, the posterior leaflets has a shorter radial length compared to the anterior leaflet (Figure 2). The posterior leaflet is also divided into lateral (P1), central (P2), and medial scallops (P3) just like the anterior leaflet. However, contrary to anterior leaflet it does have true slits within its tissue demarcating these scallops (see Figure 2) (15).

Commissural leaflets are composed of additional leaflet tissue found at the anterolateral (A1-P1) and posteromedial (A3-P3) commissures (see Figure 2) (12).

Histologically, the mitral leaflet tissue has three layers, including the fibrosa (on the ventricular surface), spongiosa (the mid layer), and atrialis. Endothelial cells cover the blood-interfacing surfaces on both atrial and ventricular surfaces. Each tissue layer has unique matrix characteristics. The fibrosa, that has to withstand the higher LV pressures, is composed of dense collagen, improving mechanical stability. The spongiosa has less organized collagen, but is rich in hydrophilic proteins at the tips ensuring a tight seal. The atrialis contains a rich network of collagen and elastin that may play a role in leaflet remodeling and adaptation (16). In both leaflets, cardiac muscle cells are present close to the annulus. This muscle tissue is excitable from the atrial side, apart and before LV excitation, and histologically resemble atrial myocardial calls (17). These muscle cells may contract even before the beginning of LV contraction and may play a role in the early closure of mitral leaflets observed before ventricular contraction. This early “leaflet” contraction may be important for avoiding early systolic regurgitation during the iso-volumic contraction period. Interstitial cells in leaflets are usually inactive, and their turnover is slow. Nevertheless, physiologic- or pathologic- induced leaflet stress can induce interstitial cell activation and proliferation (16). This proliferation suggests a potent adaptation mechanism, inducing leaflet augmentation during pathologic conditions associated with annular dilatation. Although both leaflets have three layers, their microstructure differs significantly. The anterior leaflet has to withstand a higher load and is composed mostly out of the fibrosa layer. On the other hand, the posterior leaflet is thinner and more flexible (18).

The anterior leaflet anterior also has an especially dense innervation compared with the posterior leaflet (19).

### Chordae Tendineae

The chordae tendineae are fibrous cords originating from the papillary muscle (PM) tips that insert in a hand-held fan pattern into the ventricular aspects of the anterior, posterior, and commissural leaflets (see Figure 2) (20). Rarely, chordae emanate from the basal posterior segments of the left ventricle and attach directly into the posterior leaflet. There are two main types of chordae that can be differentiated based on leaflet insertion. The primary chords attach to the leaflet-free edges. The secondary chords attach to the anterior leaflet rough zone and throughout the posterior leaflet body (21). The Chords are made of tight collagen and elastin network that distribute chordal forces over the leaflet surface (20, 21). Primary chordae are thinner and have limited extensibility that prevents leaflet edge inversion and flail (22). On the other hand, secondary chordae are thicker, and have more elastin, making them more extensible. Chordal anatomy and branching patterns is extremely variable. There is very little data concerning the posterior chords, but for the anterior chords average length and thickness are ~2 cm and 1–2 mm, respectively. Similar to the leaflets, the chords have the ability to adapt, and lengthen in response to altered loading conditions (21).

### Papillary Muscles

The lateral and medial PM are categorized based on their relationship to the mitral commissures. The bodies of PM stem from the apical one-third of the LV wall. The Chords emanate from the PM tips to the corresponding anterior, posterior, and commissural leaflet (Figure 2). The lateral PM usually has a single head. However, it has a dual blood supply from the left circumflex and left anterior descending artery. On the other hand, the medial PM usually has two heads and is either supplied by the right or circumflex coronary artery (12). PM contraction aims to control the distance between the mitral annulus and the PM tips. On one hand, early during systole longitudinal contraction of the LV base moves the entire PM (base and tip) closer to the annulus. On the other hand, later during systole, isolated PM contraction shortens the length of the papillary muscles, and increases the distance between the PM tip and the annulus. During the first half of systole papillary muscles move closer together and move concurrently toward the mitral annulus due to unopposed longitudinal contraction of the LV base. Because the mitral leaflets moves upwards toward the atrium at the same time this papillary muscle coordinated and symmetric motion, maintains equal distances between the papillary muscle tips and leaflets, avoiding distortion of mitral leaflets. Furthermore, at the same time annular contraction and folding occurs allowing early systolic co-aptation by the early saddle-shape accentuation. At the mid and late systolic period the PM bodies contract, and PM tip are pulled downwardly, away from the annulus and closing leaflets, keeping both leaflets under directed tension and posterior restrain to prevent systolic anterior motion of the leaflets, and to avoid left ventricular outflow tract obstruction by the sail-like anterior leaflet.

### Pathophysiology of MR

**Figure 3:**
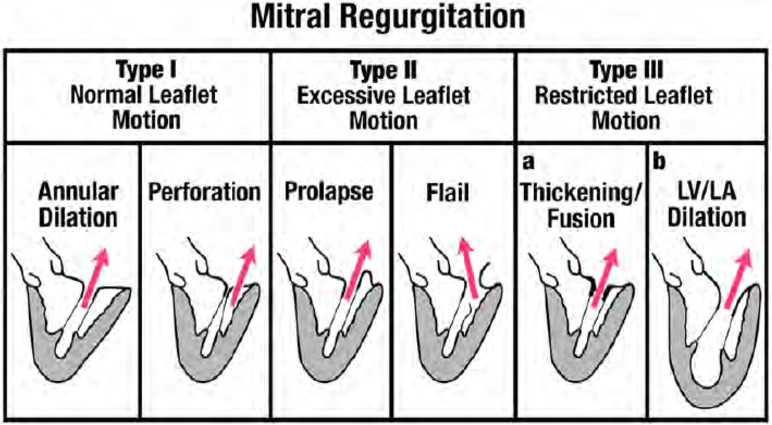
Depiction of mechansims of MR based on the Carpentier classification. From Zoghbi et al. Recommendations for noninvasive evaluation of native valvular regurgitation, A report from the American Society of Echocardiography developed in collaboration with the society for cardiovascular magnetic resonance. Journal of the American society of echocardiography April 2017; 304–363; with permission (33).

The basic mechanisms for MR were described by Carpentier and are based on the mobility of the leaflets (Figure 3). Type I MR involves normal mobility of leaflets with poor co-aptation due to annular dilation or perforation of a leaflet. Classic etiologies associated with the first mechanism (annular dilatation) are MR due to enlarged left atrium usually associated with chronic atrial fibrillation. Etiologies associated with the second mechanism (leaflet perforation) include endocarditis, iatrogenic trauma, or congenital disease. Type II MR involves excessive mobility (prolapse or ruptured chordae) of the leaflets. The classic etiologies associated with these mechanisms are mitral valve prolapse for the first, and fibro-elastic deficiency for the latter. The third mechanism (Type III) is associated with attenuated mobility of leaflets, resulting in co-aptation of leaflets in the ventricular level. This attenuated mobility can be diastolic and systolic (Type IIIa) or just systolic (Type IIIb). The first type (Type IIIa) is secondary to shrinkage of leaflets and/or chords due to inflammatory or congenital disease. Classic etiologies associated with this mechanism are rheumatic heart disease, Carcinoid, or radiation induced MR. In Type IIIb the attenuated mobility is entirely systolic and is always associated with LV enlargement, displacement of papillary muscles away from the mitral annulus, and systolic tethering of mitral leaflets transferred through the tensed chordae tendineae.

### Type I MR

**Figure 4:**
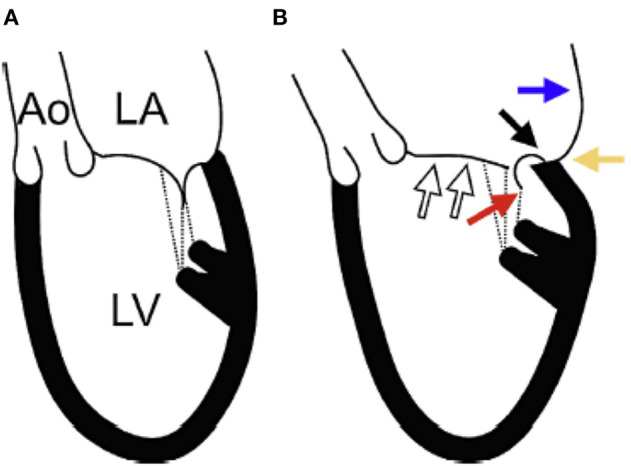
The etiology of atrial functional MR. **(A)** Schematic representation of a normal heart. **(B)** Schematic representation of a heart with Type I functional MR as demonstrated in patients with atrial fibrillation. (White arrows): the anterior mitral leaflet is flattened along the mitral annular plane with left atrial (LA) dilation and associated mitral annular dilation. (Blue arrow): the posterior wall of the LA extends behind the posterior mitral annulus due to LA dilation. (Black arrow): the posterior mitral annulus is displaced backward and upwards to the LA side from the crest of the posterior left ventricle (LV). (Yellow arrow): LA enlargement leads to the inward bending of the basal posterior LV. (Red arrow): the tip of the posterior mitral leaflet is tethered toward the posterior LV by the papillary muscles and the chordae tendineae. As a result, the posterior mitral leaflet curves, and its movement becomes restricted. Ao, ascending aorta; LA, left atrium; LV, left ventricle. From Ito et al. (26) Mechanism of atrial functional mitral regurgitation in patients with atrial fibrillation: A study using three-dimensional transesophageal echocardiography. Journal of Cardiology 70 (2017) 584–590 with permission.

**Figure 5:**
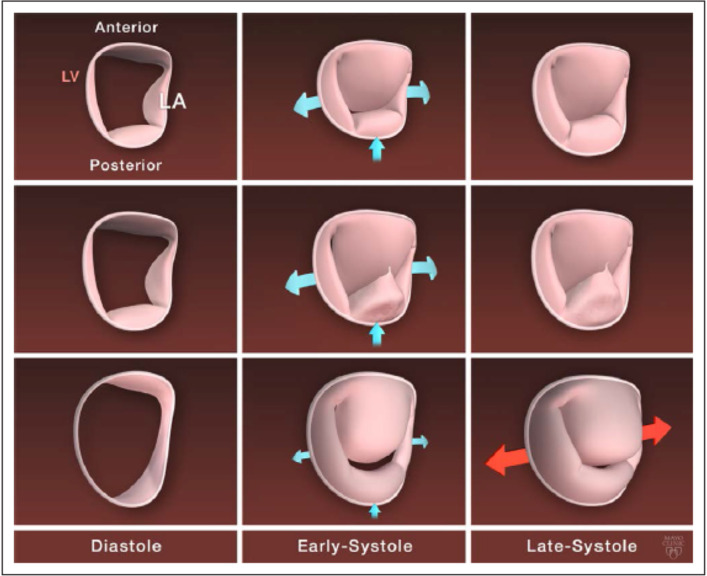
Mitral annulus dynamic in normal mitral valve and by degenerative mitral regurgitation phenotype. Top row, Normal mitral annulus dynamic with stretching of the commissure toward the ventricle and anteroposterior contraction in early systole and few modification in late systole. Middle row, Mitral annulus dynamic in fibroelastic deficiency: moderately enlarged dynamic with similar motion in early systole but of decreased magnitude compared with normal annulus. Bottom row, Mitral annulus dynamic in diffuse myxomatous disease with a severely enlarged and flattened mitral annulus, a severe decrease in the anteroposterior contraction in early systole, and an abnormal enlargement of the intercommissural diameter in late systole. LA indicates left atrial; and LV, left ventricle. From Antoine et al. (34) pathophysiology of degenerative mitral regurgitation. Circulation: cardiovascular imaging. 2018; 11 with permission.

Type I MR involves normal mobility of leaflets with poor co-aptation due to annular dilation or perforation of a leaflet. In this review we will focus on Type I functional MR due to pure annular dilatation because it is the most puzzling and poorly understood of all types. Usually, mitral annular dilatation does not result in significant MR because it is counterbalanced by the fact that LV papillary muscles provide chords to both MV leaflets and “hold them together.” Furthermore, in the context of AF, the better-developed fibrous skeleton of the mitral annulus rarely advances to severe dilatation compared with the less developed and thinner tricuspid annulus. Thus, severe tricuspid regurgitation is much more common than severe MR in patients with atrial fibrillation (23). However, when massive atrial enlargement occurs, usually due to chronic prolonged atrial fibrillation, the resulting extensive annular dilatation may overwhelm these protective mechanisms and may result in severe functional MR without systolic restrictive motion and tethering of leaflets. By studying follow-up echocardiograms after AF ablation, Gertz et al. (24) evaluated the pathophysiological mechanisms underlying “pure” annular dilatation secondary to atrial fibrillation resulting in functional Type I MR. They showed that patients with successful ablations experienced significant reductions in LA size and mitral annular dimension, and only less than a third still had still significant MR at follow-up. In contrast, among patients who had recurrence of AF, there was no significant change in annular dimension despite reductions in LA size. Over 80% of the patients with recurrence still had significant MR at follow-up. Their findings showed that mitral annular dimension was the only independent echocardiographic predictor of MR. Another possible mechanism for development of MR in patients with atrial fibrillation involves the loss of LA function. Well-timed atrial contraction at end diastole is followed by atrial relaxation which may “suck in” the mitral leaflets and be important for appropriate mitral valve closing (25). In patients with atrial fibrillation there is neither atrial contraction, nor atrial relaxation, thus the atrial mechanism for mitral closure is lost. In patients with Type I functional MR the LV is generally not significantly dilated and LV systolic function appears normal. This type of MR occurs mostly in elderly patients (~80 years old). Recently, a group from Japan tried to clarify the mitral geometric changes in patients with atrial functional MR using 3D echocardiography (26). They described the following changes: (1) LA dilatation; (2) MA dilatation; (3) the LV basal posterior wall was bent inward; (4) the anterior mitral leaflet was flattened along the mitral annular plane; and (5) the posterior mitral leaflet was bent toward the LV cavity (**Figure 5**). The authors postulated that the posterior leaflets bending seen in the patients with Type I MR due to LA dilatation had the same mechanism previously described in patients with giant LA due to mitral stenosis. In patients with giant LA, the posterior wall of the LA extends behind the basal posterior wall of the LV. The backward LA enlargement leads to the inward bending of the basal posterior LV, and the tip of the posterior leaflet is tethered to the posterior LV by the papillary muscles and its chords. This atriogenic tethering of the posterior leaflet results in a reduction of co-aptation and worsening MR (Figure 4).

Many unresolved questions remain in this type of MR: Is it associated with excess mortality despite normal LV systolic function?: Will it l improve with simple annuloplasty or mitral replacement?: What is the reason for LA dilatation in patients in sinus rhythm?; And should we treat moderate MR when the patients require tricuspid surgery for severe regurgitation due to right-sided annular enlargement secondary to chronic atrial fibrillation. It seems imperative to focus future research on all these questions before we start implanting devices without understanding what it is we treat and what is the degree of benefit.

### Type II MR

Type II MR involves excessive mobility (prolapse or ruptured chordae) of the leaflets which can affect one or both leaflets and one or multiple scallops. Two different phenotypes are described: fibroelastic deficiency (FED) and myxomatous disease (also called Barlow disease). FED is mostly localized to one segment, involves ruptured chords with leaflet redundancy and thickening mostly only on the flail segment (usually posterior, especially P2). Usually the remainder of the valve is thin and normal. Conversely, myxomatous disease (MD) usually shows generalized redundancy and thickening of both leaflets, involving multiple segments (Figure 3). In MD, MR is typically mostly in mid late systole while it is usually holo-systolic in FED. It is still uncertain whether these diseases are variants along a single pathophysiologic spectrum but recent data showing distinct physiological differences suggests that FED and MD are related but separate entities.

#### Mitral Annulus Dynamics

MA in patients with MD is enlarged, flattened, and more circular, with increased anteroposterior diameter, inter-commissural diameter, circumference, and area compared with normal valves. On the other side, annulus height is close to normal. Annular enlargement is correlated with severity of MR (27, 28). Importantly, annular enlargement in patients with MD is different from that of patients with ischemic MR, in which only the anteroposterior annulus is enlarged. In Type II MR there is also marked inter-commissural enlargement, suggesting that annular enlargement is an intrinsic part of the disease (1, 29).

The dynamics of Type II MR annular are controversial. In some studies (4) normal annular dynamics have been described.

Conversely, we and others (28, 29) observed abnormal early-systolic annular dynamics in patients with Type II MR. These patients had reduced anteroposterior contraction combined with simultaneous enlargement of inter-commissural diameter resulting in diminished annular area contraction. Systolic saddle shape accentuation was delayed and attenuated. Because early systolic saddle shape accentuation seems to play a role in avoiding early systolic regurgitation, these abnormalities can result in the addition of an early-systolic regurgitation component in patients with mitral prolapse and may lead to severe holo-systolic regurgitation. Furthermore, in late systole the annulus increases in area instead of the normal decrease, which may contribute to further separation of late systolic mitral leaflets and accentuation of the late systolic component of MR (Figure 5 and Supplementary Movies 2A,B). Some differences between annular dynamics in patients with FED vs. MD raise the question if Type II MR should be seen as a single entity or as different dynamic phenotypes (30). For similar MR severity and left ventricle, or left atrium dimensions, the annulus in patients with MD was larger, flatter, and with more inter-commissural enlargement than in patients with FED (Figure 5). This exaggerated annular enlargement in patients with MD was unexplained by LV or LA remodeling, or by the severity of regurgitation suggesting that it is an intrinsic part of the disease and not just secondary to MR or its consequences. On the other side, in patients with FED annular motion and dimensions were close to normal (Figure 5) and correlated with MR severity. In conclusion, it seems that in patients with Barlow's disease annular dynamics are extremely abnormal as an intrinsic part of the disease and play a significant role in perpetuating MR. On the other side, in FED the primary disease is of leaflets and annular dysfunction and dilatation is just secondary to MR.

#### Leaflet in Type II MR

Leaflet redundancy could not be quantified until new 3D echo technology allowed direct measurement of leaflets' areas. Recent reports have shown that in patients with Type II MR leaflet tissue area is increased vs. controls. Furthermore, prolapse volume and height were correlated with the severity of MR (27, 31). Interestingly, for similar severity of MR, leaflet redundancy is larger in MD vs. FED (31). Importantly, dynamic differences between patients with MD and FED were even more remarkable. In patients with MD larger dynamic increase of prolapse volume was seen compared to patients with FED. In patients with FED leaflets' areas remain stable throughout the cardiac cycle. However, in MD leaflet's prolapse volume and height increases in late systole. This suggests that there is very little valvular tissue reserve in FED compared to substantial tissue reserve available to expand during systole in MD (30). Figure 5 summarizes the differences between FED and MD phenotypes of Type II MR from static and dynamic perspectives.

#### Type III MR

The third mechanism (Type III) for MR is associated with decreased mobility of leaflets, resulting in co-aptation of leaflets in the ventricular level (Figure 3). This attenuated mobility can be diastolic and systolic (Type IIIa) due to organic shrinkage of leaflets and /or chords, or just systolic (Type IIIb). In this review we will focus on Type IIIb MR due to systolic restricted movement of leaflets secondary to LV systolic dysfunction and adverse remodeling.

Type IIIb functional MR occurs despite structurally normal mitral leaflets as a consequence of LV dysfunction. It is unquestionably associated with LV remodeling and enlargement. It has been attributed to global LV dilatation, mitral annulus enlargement, or local LV remodeling associated with apical and posterior displacement of papillary muscles leading to excess valvular tenting (32).

Recent advances in 3D Doppler echocardiography allow assessment of regurgitant flow throughout the cardiac cycle. These advances permit new insights into the pathologic dynamics of the mitral annulus, and papillary muscle movement (1).

#### Mitral Annulus Dynamics in Type III MR

Compared with control subjects, the annulus in patients with Type IIIb MR is larger throughout the cardiac cycle. We have recently shown that although inter-commissural diameter was similar in all patients with low ejection fraction (EF) with or without MR, patients with Type IIIb MR have a larger antero-posterior diameter compared to patients with systolic dysfunction but no MR. Furthermore, not only baseline annular geometry was different, but also annular dynamics were pathologic in patients with Type IIIb MR. In these patients early systolic annular folding, and saddle-shape deepening was absent. In fact in patients with significant Type IIIb MR an adynamic annulus in terms of saddle shape was observed.

In conclusion, the loss of annular folding across the inter-commissural axis and the loss of saddle shape accentuation in early systole, plays a role in early-systolic Type IIIb MR just as it is in myxomatous mitral valve disease (1).

#### Papillary Muscles Type IIIb MR

In patients with low EF, irrespective of MR severity, inter-papillary muscle approximation is attenuated. This is possibly due to reduced circumferential and radial basal contraction. On the other hand, there are marked differences between patients with and without FMR, in terms of papillary muscle movement toward the mitral annulus. In patients with low EF but no MR papillary muscle to mitral annulus approximation is suppressed compared to control patients, but the distances between the two muscles and the annulus remain equal and symmetric, avoiding excessive mid-systolic tethering and distortion of mitral leaflets. In contrast, in patients with Type IIIb MR postero-medial muscle tip tends to paradoxically move away from the annulus during mid systole. Thus, as opposed to the normal valve, irrespective of EF, in which firm apposition of the leaflets by intra-ventricular pressure is assisted by the symmetric descent of papillary muscles toward the annulus, in patients with Type IIIb MR, asymmetric mid-systolic papillary muscle displacement results in abnormal tethering geometry (1).

In conclusion, as opposed to early-systole, in which annular folding plays a critical role, in the second and third parts of systole, asymmetric papillary muscle motion toward the annulus is the main determinant of late systolic MR.

#### Subgroup Analysis

Analysis of the mechanism of MR in patients with anterior MI, global dysfunction and inferior MI shows that those mechanisms differ depending on the etiology of LV dysfunction (1). In patients with anterior MI and/or global dysfunction the loss of normal deepening of saddle shape contributes significantly to worse early systolic MR, but it is less important in patients with inferior MI. On the other hand, in patients with inferior MI early systolic MR depends entirely on tenting volume. On the other hand, in mid-systole MR depends only on annular area in patients with anterior MI, but on symmetry and coordination of papillary muscle motion in both inferior MI and global dysfunction patients.

#### Mitral Valve Repair

Mitral valve repair has become the procedure of choice in patients with functional MR because of its advantages over valve replacement regarding long-term survival and freedom from valve-related adverse events. In the early seventies Carpentier initiated the modern era of mitral valve repair (35). Since then the repair techniques have continued to evolve with the improved understanding of the structure and dynamics of the mitral valve. In this paragraph we will briefly summarize the standard surgical corrections for the most common mechanisms of MR.

#### Type II MR

The most common mechanism of MR needing mitral repair is Type II MR. Carpentier was the first to introduce the quadrangular resection technique, which became the first standard to treat posterior leaflet prolapse (35). In this technique the first step is to identify the prolapsing segment of the mitral leaflet and excise it with its associated ruptured or elongated chordae from the free margin of the leaflet down to the annulus. In the second step annular plication is performed to reduce the orifice size. The repair is completed by an annuloplasty band or ring. In the following years the triangular resection technique was developed to simplify the operation and reduce the risk of complications by eliminating the need for annular plication (36). This technique is ideal for patients with segmental posterior leaflet prolapse but can also be used in isolated anterior prolapse involving a small segment as well. To avoid systolic anterior motion (SAM) the “sliding plasty” technique was added to reduce the height of the posterior leaflet to prevent SAM. This technique is useful mostly for patients with Barlow's disease or in cases with excess leaflet tissue of the prolapsing posterior segment (35). Since then numerous other techniques to avoid SAM have been introduced. Although resection techniques are associated with favorable outcomes, they have some drawbacks including reduction of leaflet tissue, which is the major component of the surface of coaptation and the need for annulus plication that may deform of the sub annular region of the left ventricle resulting in injury to circumflex artery or SAM. Frater and David introduced the use of artificial chordae to replace elongated or ruptured chordae responsible for mitral valve prolapse and mitral regurgitation (37, 38). These neo-chordae used polytetrafluoroethylene (PTFE) sutures, usually made of Gore-Tex. The neo-chordae eliminate prolapse by supporting the free edge of the leaflet and thereby produce an optimal surface of coaptation. Usually the PTFE suture is passed through the fibrous region of a papillary muscle on the same side of the valve as the region of prolapse. Each end of the suture is brought up to the leaflet edge and passed through the leaflet tissue in the region of prolapse. In the final stage the chordal length is adjusted to a level to prevent prolapse and the suture is tied on the atrial side of the leaflet (37, 38). Another common technique, the edge to edge repair was introduced by Alfieri et al. in the early nineties. This technique is especially useful for correction of isolated anterior leaflet prolapse or bileaflet prolapse due to Barlow's disease. This approach involves anchoring the free edge of the prolapsing leaflet to the corresponding free edge of the opposite leaflet, resulting in a double orifice valve. An annuloplasty completes the repair (39). Due to the risk of inducing stenosis, the edge-to-edge repair is not recommended in patients with a small mitral valve area. Furthermore, results are sub-optimal when an annuloplasty is not included. Finally, poor results after anterior leaflet resection led to a variety of repair methods including chordal transfer, chordal shortening and artificial chordal replacement. Because the use of neochordae has been associated with improved results as compared to chordal transfer, it is currently the procedure of choice to correct anterior leaflet prolapse (37, 38).

#### Type IIIb MR

After Type II MR, Type IIIb is the most common mechanism for mitral valve repair. Mitral valve repair with a restrictive annuloplasty has been the treatment of choice to address Type IIIb MR for many years. It restores leaflet coaptation by decreasing the anteroposterior distance and the valve area (40). The annuloplasty is performed with a complete and rigid ring at least one or two sizes smaller than the size necessary to improve leaflet coaptation. In some cases LV remodeling may continue after repair resulting in further displacement of the papillary muscles worsening tethering and recurrence of MR. Because the rate of MR recurrence after restrictive annuloplasty may reach 10–20% rates early after operation and up to 50–70% at 5 years multiple annuloplasty rings have been designed to address the changes in annular shape associated with Type IIIb MR. However, there are no studies that prove their superiority. Other adjuvant techniques, include division of secondary chordae, placement of edge to edge stitches, and reposition of papillary muscles, have been promoted to decrease the rate of recurrence (40). However, long term results and indications for those techniques are still lacking.

#### Type I MR

Type I functional MR is rare and is usually repaired by annuloplasty. A number of annuloplasty devices are available and include complete (rings) or partial (bands), and may be rigid, semi-rigid or flexible. Currently there is no consensus regarding the selction of annuloplasty device.

While surgery remains the mainstay for treatment in MR, several technological advances in the last years have made trans-catheter mitral valve interventions feasible and safe. The use of these techniques in patients with severe MR has shown promise in reducing symptoms, improving quality of life, with potential for a survival advantage among certain patients with secondary MR. The most commonly used device is the MitralClip (Abbott Laboratories, Menlo Park, California, USA), a cobalt chromium clip covered that has two arms and works by grasping and approximating edges of the anterior and posterior valvular leaflet segments. The MitralClip was designed after the surgical Alfieri technique and received CE-Mark and FDA approval for use in primary Type II MR and functional Type I and IIIb MR. Another similar device is the PASCAL TMVR (Edwards Lifesciences, Irvine, CA). Other devices include the indirect annuloplasty devices such as the Carillon system, ARTO device, Mitral Loop Cerclage Catheter System, and the direct annuloplasty devices such as the Cardioband (Edwards Lifesciences, Irvine, CA), Mitralign system (Mitralign, Tewksbury, Massachusetts) and the Millipede IRIS ring, all imitating surgical annuloplasty and designed to treat functional Type I and IIIb MR.

## Conclusions

MR is affected by a complex dynamic change of the annulus, leaflets, chords, papillary muscles, and atrial and ventricular interaction. It is associated with different mechanism depending of the Type of MR. In patients with Type I MR it is linked mostly to annular and left atrial factors. In patients with Type II MR, the major contributors are related to annular and leaflets factors. In Type IIIb MR the major contributors are tenting volume, loss of annular contraction across the inter-commissural axis (annular folding), and to asymmetric papillary muscle dynamic changes that are linked to the valve deformation. These new insights should lead to refined concepts for MR pathophysiology and repair techniques.

## Summary

In this review we describe the anatomy and physiology of the different parts of the mitral valve apparatus including the annulus, leaflets, chordae tendineae and papillary muscles in normal subjects and with different types of mitral regurgitation (MR). We show that MR is affected by a complex dynamic change of all the components of the apparatus and their interactions. Furthermore, different types of MR are associated with different mechanisms and pathologic interactions. Type I MR it is linked mostly to annular and left atrial factors, Type II MR is related mostly to annular and leaflets factors but in Type IIIb the major contributors are tethering of leaflets, loss of annular folding, and asymmetric papillary muscle dynamic changes that are linked to the valve deformation. These new insights should lead to refined concepts for MR pathophysiology and repair techniques.

## Author Contributions

The author confirms being the sole contributor of this work and has approved it for publication.

## Conflict of Interest

The author declares that the research was conducted in the absence of any commercial or financial relationships that could be construed as a potential conflict of interest.

## Supplementary Material

Supplementary Movie 1 Schematic representation of a 3D reconstructed normal mitral annulus and leaflets in motion. Note that in early-systole anteroposterior folding across the fixed inter-commissural diameter occurs, leading to early-systolic annular area contraction, accentuation of saddle shape, and approximation of anterior, and posterior leaflets. This annular contractions results in leaflet approximation when systolic ventricular pressure is still relatively low and does not yet press the leaflets together and may be important in preventing early systolic mitral regurgitation.

Supplementary Movie 2 **(A,B)** Schematic representation of a 3D reconstructed mitral annulus and leaflets (oblique view **A**; En face **B**) in motion in a patient with Type II MR compared to normal valve. Note that in normal valve the mitral annulus is dynamic with anteroposterior contraction and accentuation of saddle shape in early systole. On the other hand, the mitral annulus in myxomatous disease is severely enlarged and flattened and has an attenuated anteroposterior contraction in early systole, and an abnormal enlargement of the inter-commissural diameter in late systole.
